# Bis[4-(1-imino­eth­yl)-3-methyl-1-phenyl-1*H*-pyrazol-5-olato-κ^2^ *O*,*N* ^4^]copper(II)

**DOI:** 10.1107/S1600536811030753

**Published:** 2011-08-06

**Authors:** Hualing Zhu, Zhan Wang, Zhen Wei, Yanan Bai, Xiaoping Xv

**Affiliations:** a Department of Basic Science, Tianjin Agriculturial College, Tianjin Jinjing Road No. 22, Tianjin 300384, People’s Republic of China

## Abstract

In the title complex, [Cu(C_12_H_12_N_3_O)_2_], the Cu^II^ ion is tetra­coordinated by two N atoms and two O atoms from two bis-chelating 4-(1-imino­eth­yl)-3-methyl-1-phenyl-1*H*-pyrazol-5-olate ligands in a square-planar geometry. The two N atoms and two O atoms around the Cu^II^ atom are *trans* to each other, as the Cu^II^ atom lies on an inversion centre. The six-membered ring composed of the Cu, an O, an N and three C atoms of the ligand and the pyrazole ring is nearly planar, the largest deviation being 0.037 (4) Å for an N atom. In the crystal, weak inter­molecular C—H⋯N hydrogen-bonding inter­actions link the mol­ecules into chains along the *c* axis.

## Related literature

For our ongoing studies on pyrazolone derivatives, see: Zhu, Shi *et al.* (2010 ▶); Zhu, Wei *et al.* (2010 ▶). For related structures, see: Parsons *et al.* (2004 ▶); Shi *et al.* (2005 ▶).
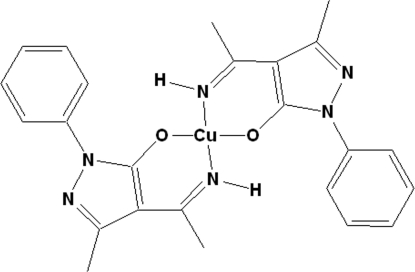


## Experimental

### 

#### Crystal data

[Cu(C_12_H_12_N_3_O)_2_]
*M* *_r_* = 492.03
Monoclinic, 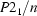

*a* = 6.391 (6) Å
*b* = 9.010 (8) Å
*c* = 18.772 (17) Å
β = 98.701 (17)°
*V* = 1068.5 (16) Å^3^
*Z* = 2
Mo *K*α radiation
μ = 1.06 mm^−1^
*T* = 113 K
0.10 × 0.10 × 0.10 mm

#### Data collection

Rigaku Saturn724 CCD diffractometer
Absorption correction: multi-scan (*CrystalClear*; Rigaku/MSC, 2008) ▶ *T*_min_ = 0.902, *T*_max_ = 0.902
8871 measured reflections
1888 independent reflections
1636 reflections with *I* > 2σ(*I*)
*R*_int_ = 0.130

#### Refinement

*R*[*F* ^2^ > 2σ(*F* ^2^)] = 0.063
*wR*(*F* ^2^) = 0.136
*S* = 1.08
1888 reflections
154 parameters
H-atom parameters constrained
Δρ_max_ = 0.62 e Å^−3^
Δρ_min_ = −1.64 e Å^−3^

### 

Data collection: *CrystalClear* (Rigaku/MSC, 2008 ▶); cell refinement: *CrystalClear*; data reduction: *CrystalClear*; program(s) used to solve structure: *SHELXS97* (Sheldrick, 2008 ▶); program(s) used to refine structure: *SHELXL97* (Sheldrick, 2008 ▶); molecular graphics: *SHELXTL* (Sheldrick, 2008 ▶); software used to prepare material for publication: *CrystalStructure* (Rigaku/MSC, 2008 ▶).

## Supplementary Material

Crystal structure: contains datablock(s) I, global. DOI: 10.1107/S1600536811030753/pv2436sup1.cif

Structure factors: contains datablock(s) I. DOI: 10.1107/S1600536811030753/pv2436Isup2.hkl

Additional supplementary materials:  crystallographic information; 3D view; checkCIF report

## Figures and Tables

**Table 1:** Hydrogen-bond geometry (Å, °)

*D*—H⋯*A*	*D*—H	H⋯*A*	*D*⋯*A*	*D*—H⋯*A*
C11—H11⋯N1^i^	0.95	2.61	3.366 (6)	137

Symmetry code: (i) 
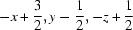
.

**Fig. 1.**
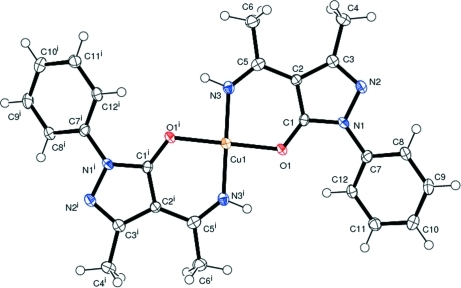
The molecular structure of the title compound with the atom numbering scheme. Displacement ellipsoids are drawn at the 30% probability level. Symmetry code: i: -x + 1/2, -y + 1/2, -z + 1/2).

**Fig. 2.**
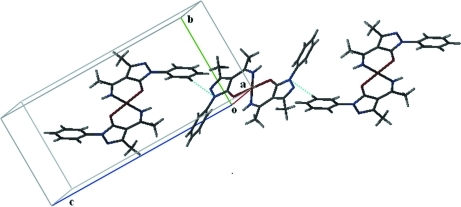
Part of the crystal structure showing intermolecular hydrogen bonding interactions (C—H···N) as dashed lines.

## supplementary crystallographic information

### Comment

As a part of our onging studies on pyrazolone derivatives as potential ligands (Zhu, Shi *et al.*, 2010; Zhu, Wei *et al.*, 2010) we report the structure of the title complex in this article.

In the title complex (Fig. 1.), the central Cu^II^ ion is tetracoordinated by two N atoms and two O atoms from two bis-chelating 4-(2-methyl iminomethyl)-3-methyl- 1- phenyl-1*H*-pyrazol-5(4*H*)-onato ligands in a square-planar geometry. The two N atoms and two O atoms around the Cu^II^ atom are *trans* to each other, as the Cu^II^ atom lies on an inversion centre. The six membered chelate ring (Cu1/O1/N3/C1/C2/C5) / and the pyrazol ring are nearly coplannar with the largest deviation 0.037 (4)Å for N2 atom. In the crystal structure, weak intermolecular hydrogen bonding interactions (C11—H11···N1) link molecules into one-dimensional chains (Fig. 2).

### Experimental

The title compound was synthesized by dropping a copper acetate (15 mmol) ethanolic solution into an ethanolic solution of 4-[(3,4-dihydro-5-methyl-3-oxo-2-phenyl-2*H*-pyrazol-4–ylidene)(methyl) methylamino]-1,5-dimethyl-2-phenyl-1*H*-pyrazol-3(2*H*)-one(30 mmol) and stirring for about 7 h at room temperature. The dark green blocks which were obtained were dried in air. The product was recrystallized from *N*,*N*-dimethylformamide which afforded crystals suitable for *X*–ray analysis.

### Refinement

The H atoms were geometrically positioned and refined using a riding model, with N—H = 0.88 Å and C—H = 0.95 or 0.98 Å for the aryl or methyl H atoms, respectively, and *U*_iso_(H) = 1.2 *U*_eq_(N/C-aryl) or 1.5*U*_eq_(C-methyl).

### Crystal data

**Table:**

[Cu(C_12_H_12_N_3_O)_2_]	*F*(000) = 510
*M**_r_* = 492.03	*D*_x_ = 1.529 Mg m^−^^3^
Monoclinic, *P*2_1_/*n*	Mo *K*α radiation, λ = 0.71073 Å
Hall symbol: -P 2yn	Cell parameters from 3203 reflections
*a* = 6.391 (6) Å	θ = 2.2–27.9°
*b* = 9.010 (8) Å	µ = 1.06 mm^−^^1^
*c* = 18.772 (17) Å	*T* = 113 K
β = 98.701 (17)°	Block, dark green
*V* = 1068.5 (16) Å^3^	0.10 × 0.10 × 0.10 mm
*Z* = 2	

### Data collection

**Table:**

Rigaku Saturn724 CCD diffractometer	1888 independent reflections
Radiation source: rotating anode	1636 reflections with *I* > 2σ(*I*)
multilayer	*R*_int_ = 0.130
Detector resolution: 14.22 pixels mm^-1^	θ_max_ = 25.0°, θ_min_ = 2.2°
ω and φ scans	*h* = −7→7
Absorption correction: multi-scan (*CrystalClear*; Rigaku/MSC, 2008)	*k* = −10→10
*T*_min_ = 0.902, *T*_max_ = 0.902	*l* = −22→21
8871 measured reflections	

### Refinement

**Table:**

Refinement on *F*^2^	Primary atom site location: structure-invariant direct methods
Least-squares matrix: full	Secondary atom site location: difference Fourier map
*R*[*F*^2^ > 2σ(*F*^2^)] = 0.063	Hydrogen site location: inferred from neighbouring sites
*wR*(*F*^2^) = 0.136	H-atom parameters constrained
*S* = 1.08	*w* = 1/[σ^2^(*F*_o_^2^) + (0.043*P*)^2^ + 2.9195*P*] where *P* = (*F*_o_^2^ + 2*F*_c_^2^)/3
1888 reflections	(Δ/σ)_max_ < 0.001
154 parameters	Δρ_max_ = 0.62 e Å^−^^3^
0 restraints	Δρ_min_ = −1.64 e Å^−^^3^

### Special details

**Table:**

Geometry. All e.s.d.'s (except the e.s.d. in the dihedral angle between two l.s. planes) are estimated using the full covariance matrix. The cell e.s.d.'s are taken into account individually in the estimation of e.s.d.'s in distances, angles and torsion angles; correlations between e.s.d.'s in cell parameters are only used when they are defined by crystal symmetry. An approximate (isotropic) treatment of cell e.s.d.'s is used for estimating e.s.d.'s involving l.s. planes.
Refinement. Refinement of *F*^2^ against ALL reflections. The weighted *R*-factor *wR* and goodness of fit *S* are based on *F*^2^, conventional *R*-factors *R* are based on *F*, with *F* set to zero for negative *F*^2^. The threshold expression of *F*^2^ > σ(*F*^2^) is used only for calculating *R*-factors(gt) *etc*. and is not relevant to the choice of reflections for refinement. *R*-factors based on *F*^2^ are statistically about twice as large as those based on *F*, and *R*- factors based on ALL data will be even larger.

### Fractional atomic coordinates and isotropic or equivalent isotropic displacement parameters (Å^2^)

**Table:**

	*x*	*y*	*z*	*U*_iso_*/*U*_eq_	
Cu1	1.0000	0.0000	0.0000	0.0168 (2)	
O1	0.8267 (5)	0.0256 (3)	0.07637 (14)	0.0180 (6)	
N1	0.5337 (5)	0.1293 (4)	0.11912 (17)	0.0175 (8)	
N2	0.3649 (5)	0.2250 (4)	0.09578 (18)	0.0181 (8)	
N3	0.8317 (5)	0.1373 (4)	−0.06383 (17)	0.0174 (8)	
H3	0.8788	0.1521	−0.1049	0.018 (11)*	
C1	0.6638 (6)	0.1120 (4)	0.0678 (2)	0.0149 (8)	
C2	0.5770 (6)	0.2036 (4)	0.0094 (2)	0.0147 (8)	
C3	0.3932 (6)	0.2692 (4)	0.0303 (2)	0.0172 (9)	
C4	0.2369 (7)	0.3782 (5)	−0.0080 (2)	0.0226 (9)	
H4A	0.1279	0.4000	0.0220	0.034*	
H4B	0.1704	0.3356	−0.0540	0.034*	
H4C	0.3105	0.4701	−0.0171	0.034*	
C5	0.6613 (7)	0.2121 (4)	−0.0571 (2)	0.0186 (9)	
C6	0.5517 (7)	0.3040 (5)	−0.1183 (2)	0.0248 (10)	
H6A	0.6266	0.2943	−0.1599	0.037*	
H6B	0.5513	0.4083	−0.1035	0.037*	
H6C	0.4056	0.2694	−0.1314	0.037*	
C7	0.5388 (7)	0.0532 (4)	0.1862 (2)	0.0171 (9)	
C8	0.3507 (7)	0.0359 (5)	0.2136 (2)	0.0217 (9)	
H8	0.2236	0.0788	0.1896	0.026*	
C9	0.3505 (7)	−0.0449 (5)	0.2766 (2)	0.0242 (10)	
H9	0.2219	−0.0586	0.2953	0.029*	
C10	0.5372 (7)	−0.1061 (5)	0.3124 (2)	0.0239 (10)	
H10	0.5354	−0.1621	0.3552	0.029*	
C11	0.7253 (7)	−0.0854 (5)	0.2858 (2)	0.0220 (9)	
H11	0.8529	−0.1265	0.3105	0.026*	
C12	0.7277 (7)	−0.0043 (4)	0.2227 (2)	0.0182 (9)	
H12	0.8570	0.0117	0.2046	0.022*	

### Atomic displacement parameters (Å^2^)

**Table:**

	*U*^11^	*U*^22^	*U*^33^	*U*^12^	*U*^13^	*U*^23^
Cu1	0.0155 (4)	0.0184 (4)	0.0171 (4)	0.0023 (3)	0.0047 (3)	−0.0003 (3)
O1	0.0137 (14)	0.0223 (15)	0.0186 (14)	0.0057 (12)	0.0044 (11)	0.0013 (12)
N1	0.0137 (17)	0.0185 (18)	0.0205 (17)	0.0046 (14)	0.0034 (14)	0.0012 (14)
N2	0.0151 (17)	0.0160 (17)	0.0240 (18)	0.0013 (14)	0.0057 (14)	−0.0006 (14)
N3	0.0184 (18)	0.0208 (18)	0.0140 (16)	−0.0009 (15)	0.0055 (14)	−0.0024 (14)
C1	0.0136 (19)	0.015 (2)	0.0166 (18)	−0.0032 (16)	0.0036 (16)	−0.0009 (15)
C2	0.015 (2)	0.0124 (19)	0.0170 (19)	−0.0017 (16)	0.0030 (16)	0.0006 (16)
C3	0.017 (2)	0.0125 (19)	0.022 (2)	−0.0026 (16)	0.0029 (17)	−0.0026 (16)
C4	0.020 (2)	0.020 (2)	0.027 (2)	0.0031 (18)	0.0017 (19)	0.0018 (18)
C5	0.021 (2)	0.016 (2)	0.018 (2)	−0.0043 (17)	0.0006 (17)	−0.0016 (17)
C6	0.030 (3)	0.024 (2)	0.020 (2)	0.004 (2)	0.0029 (19)	0.0001 (18)
C7	0.023 (2)	0.015 (2)	0.0138 (18)	0.0005 (17)	0.0044 (17)	−0.0034 (16)
C8	0.016 (2)	0.027 (2)	0.023 (2)	0.0053 (18)	0.0069 (18)	0.0012 (18)
C9	0.019 (2)	0.031 (2)	0.025 (2)	−0.0016 (19)	0.0104 (18)	−0.0015 (19)
C10	0.026 (2)	0.028 (2)	0.020 (2)	−0.004 (2)	0.0087 (19)	−0.0007 (18)
C11	0.020 (2)	0.026 (2)	0.019 (2)	0.0035 (19)	0.0014 (17)	−0.0021 (18)
C12	0.015 (2)	0.019 (2)	0.022 (2)	−0.0018 (17)	0.0058 (17)	−0.0016 (17)

### Geometric parameters (Å, °)

**Table:**

Cu1—N3^i^	1.932 (4)	C4—H4C	0.9800
Cu1—N3	1.932 (4)	C5—C6	1.502 (6)
Cu1—O1	1.953 (3)	C6—H6A	0.9800
Cu1—O1^i^	1.953 (3)	C6—H6B	0.9800
O1—C1	1.290 (5)	C6—H6C	0.9800
N1—C1	1.373 (5)	C7—C8	1.385 (6)
N1—N2	1.399 (5)	C7—C12	1.395 (6)
N1—C7	1.430 (5)	C8—C9	1.389 (6)
N2—C3	1.330 (5)	C8—H8	0.9500
N3—C5	1.303 (5)	C9—C10	1.392 (6)
N3—H3	0.8800	C9—H9	0.9500
C1—C2	1.418 (5)	C10—C11	1.383 (6)
C2—C3	1.422 (6)	C10—H10	0.9500
C2—C5	1.435 (6)	C11—C12	1.395 (6)
C3—C4	1.505 (6)	C11—H11	0.9500
C4—H4A	0.9800	C12—H12	0.9500
C4—H4B	0.9800		
			
N3^i^—Cu1—N3	180.0 (2)	H4B—C4—H4C	109.5
N3^i^—Cu1—O1	86.83 (14)	N3—C5—C2	119.1 (4)
N3—Cu1—O1	93.17 (14)	N3—C5—C6	120.7 (4)
N3^i^—Cu1—O1^i^	93.17 (14)	C2—C5—C6	120.1 (4)
N3—Cu1—O1^i^	86.83 (14)	C5—C6—H6A	109.5
O1—Cu1—O1^i^	180.00 (14)	C5—C6—H6B	109.5
C1—O1—Cu1	121.1 (2)	H6A—C6—H6B	109.5
C1—N1—N2	111.8 (3)	C5—C6—H6C	109.5
C1—N1—C7	128.9 (3)	H6A—C6—H6C	109.5
N2—N1—C7	119.0 (3)	H6B—C6—H6C	109.5
C3—N2—N1	105.5 (3)	C8—C7—C12	120.7 (4)
C5—N3—Cu1	131.7 (3)	C8—C7—N1	118.4 (4)
C5—N3—H3	114.1	C12—C7—N1	120.9 (4)
Cu1—N3—H3	114.1	C7—C8—C9	119.2 (4)
O1—C1—N1	123.0 (3)	C7—C8—H8	120.4
O1—C1—C2	131.4 (4)	C9—C8—H8	120.4
N1—C1—C2	105.6 (3)	C8—C9—C10	120.5 (4)
C1—C2—C3	105.8 (3)	C8—C9—H9	119.7
C1—C2—C5	123.3 (4)	C10—C9—H9	119.7
C3—C2—C5	130.8 (4)	C11—C10—C9	120.0 (4)
N2—C3—C2	111.4 (4)	C11—C10—H10	120.0
N2—C3—C4	117.7 (4)	C9—C10—H10	120.0
C2—C3—C4	131.0 (4)	C10—C11—C12	120.0 (4)
C3—C4—H4A	109.5	C10—C11—H11	120.0
C3—C4—H4B	109.5	C12—C11—H11	120.0
H4A—C4—H4B	109.5	C11—C12—C7	119.5 (4)
C3—C4—H4C	109.5	C11—C12—H12	120.3
H4A—C4—H4C	109.5	C7—C12—H12	120.3
			
N3^i^—Cu1—O1—C1	−178.9 (3)	C1—C2—C3—C4	178.8 (4)
N3—Cu1—O1—C1	1.1 (3)	C5—C2—C3—C4	−5.6 (7)
C1—N1—N2—C3	−1.2 (4)	Cu1—N3—C5—C2	3.6 (6)
C7—N1—N2—C3	−175.0 (3)	Cu1—N3—C5—C6	−175.4 (3)
O1—Cu1—N3—C5	−2.5 (4)	C1—C2—C5—N3	−3.1 (6)
O1^i^—Cu1—N3—C5	177.5 (4)	C3—C2—C5—N3	−178.0 (4)
Cu1—O1—C1—N1	177.7 (3)	C1—C2—C5—C6	175.9 (4)
Cu1—O1—C1—C2	−1.4 (6)	C3—C2—C5—C6	0.9 (7)
N2—N1—C1—O1	−178.0 (3)	C1—N1—C7—C8	−151.2 (4)
C7—N1—C1—O1	−4.9 (6)	N2—N1—C7—C8	21.4 (5)
N2—N1—C1—C2	1.3 (4)	C1—N1—C7—C12	27.8 (6)
C7—N1—C1—C2	174.4 (4)	N2—N1—C7—C12	−159.6 (4)
O1—C1—C2—C3	178.3 (4)	C12—C7—C8—C9	−2.6 (6)
N1—C1—C2—C3	−1.0 (4)	N1—C7—C8—C9	176.4 (4)
O1—C1—C2—C5	2.3 (7)	C7—C8—C9—C10	0.9 (7)
N1—C1—C2—C5	−177.0 (4)	C8—C9—C10—C11	0.6 (7)
N1—N2—C3—C2	0.5 (4)	C9—C10—C11—C12	−0.5 (6)
N1—N2—C3—C4	−178.2 (3)	C10—C11—C12—C7	−1.1 (6)
C1—C2—C3—N2	0.3 (5)	C8—C7—C12—C11	2.7 (6)
C5—C2—C3—N2	175.9 (4)	N1—C7—C12—C11	−176.3 (4)

Symmetry codes: (i) −*x*+2, −*y*, −*z*.

### Hydrogen-bond geometry (Å, °)

**Table:**

*D*—H···*A*	*D*—H	H···*A*	*D*···*A*	*D*—H···*A*
N3—H3···O1^i^	0.88	2.47	2.670 (5)	94.
C4—H4B···N3^ii^	0.98	2.79	3.419 (6)	123.
C9—H9···N2^iii^	0.95	2.94	3.596 (6)	128.
C11—H11···N1^iv^	0.95	2.61	3.366 (6)	137.
C11—H11···N2^iv^	0.95	2.68	3.599 (6)	163.
C12—H12···O1	0.95	2.39	2.923 (5)	115.

Symmetry codes: (i) −*x*+2, −*y*, −*z*; (ii) *x*−1, *y*, *z*; (iii) −*x*+1/2, *y*−1/2, −*z*+1/2; (iv) −*x*+3/2, *y*−1/2, −*z*+1/2.
